# Occupational stress and burnout among Japanese early childhood education and care teachers: the mediating role of psychological safety

**DOI:** 10.1080/21642850.2026.2668170

**Published:** 2026-05-06

**Authors:** Yuko Matsuda, Shoko Hamada

**Affiliations:** a Institute of Human Sciences, University of Tsukuba, Tsukuba, Japan; b Faculty of Contemporary Culture, Hijiyama University, Hiroshima, Japan

**Keywords:** Burnout, early childhood education and care teachers, Japan, occupational stress, psychological safety

## Abstract

**Background:**

Early childhood education and care (ECEC) teachers in Japan experience high levels of occupational stress, placing them at significant risk of burnout. While organizational support has been explored concerning burnout, limited research has examined the role of psychological safety as a mediating factor in this context.

**Objective:**

This study investigated the mediating role of psychological safety in the relationship between occupational stress and burnout among ECEC teachers in Japan.

**Methods:**

A longitudinal data survey was conducted in early May 2023 (Time 1) and late March 2024 (Time 2), with 500 participants completing both surveys. Measures included the Brief Job Stress Questionnaire (Time 1) and Japanese versions of the Burnout and Psychological Safety Scales (Time 2).

**Results:**

Covariance structure analysis revealed that quantitative job overload had a direct positive association with only emotional exhaustion. Higher job control was associated with lower levels across all dimensions of burnout and was partially mediated by psychological safety. Supervisor support had a direct negative association with only reduced personal accomplishment, while having a significant indirect association with all burnout dimensions through psychological safety. The association of coworker support with burnout was fully mediated by psychological safety.

**Conclusion:**

These findings underscore the critical role of psychological safety in the relationship between occupational stress and burnout. Notably, they suggest that interventions to enhance psychological safety may effectively prevent burnout among ECEC teachers.

## Introduction

Occupational stress is higher among early childhood education and care (ECEC) teachers in Japan than in other professions. While the proportion of individuals with high stress among general workers was found to be 15.3%, measured using the Brief Job Stress Questionnaire (used in stress cheques for Japanese workers; Nishimura et al., 2022), it was 24.3% among ECEC teachers (Matsuda & Hamada, 2024), highlighting the seriousness of stress-related issues among ECEC teachers. According to the Organisation for Economic Co-operation and Development (2025) ‘Starting Strong Teaching and Learning International Survey’ (TALIS Starting Strong) 2024, ECEC staff in Japan reported the longest weekly working hours among participating countries. In addition, proportions of respondents who agreed with the statements ‘Children value me as an ECEC professional,’ ‘Parents value me as an ECEC professional,’ and ‘ECEC staff are valued by society’ were the lowest in Japan among the participating countries. Compared with the international average, a higher proportion of respondents in Japan reported their sources of stress as ‘excessive administrative tasks (e.g. paperwork)’ and ‘being responsible for children’s development, learning, and well-being.’ In Japan, a chronic shortage of ECEC personnel, increased workload and responsibilities placed on practitioners, and dissatisfaction with compensation and social recognition are interrelated. This has in turn led to the emergence of issues such as inappropriate childcare practices within ECEC settings.

Such chronic stress experienced at work may lead to burnout, particularly in interpersonal helping professions such as teaching (Capel, 1992; McCormick & Barnett, 2011), healthcare (Fothergill et al., 2004; Yates, 2020), and caregiving (Grey-Stanley & Muramatsu, 2011). Burnout among ECEC teachers has been widely studied in Japan (Kiso, 2016) and internationally (De Stasio et al., 2017; Rentzou, 2015). Further, ECEC teachers report both a sense of fulfilment and risk of emotional exhaustion because of their close involvement with children, parents, and colleagues.

### Definition of burnout

Burnout is defined as ‘a prolonged response to chronic emotional and interpersonal stressors on the job’ (Maslach, 2003, 189) or ‘physical and mental symptoms expressed when one cannot cope with excessive and persistent stress, causing a relaxation of previously sustained tension and a rapid decline or lack of motivation and ambition’ (Kubo & Tao, 1992, 361).

The most commonly used measure of burnout is the Maslach Burnout Inventory, which conceptualises burnout through three symptoms (Maslach & Jackson, 1981, 99):

(1) emotional exhaustion, a state in which ‘workers feel they are no longer able to give of themselves at a psychological level’,
(2) depersonalisation, ‘the development of negative, cynical attitudes and feelings about one’s clients (service recipients),’ and
(3) reduced personal accomplishment, ‘the tendency to evaluate oneself negatively, particularly with regard to one’s work with clients.’

### Outcomes of burnout

Generally, burnout is related to reduced performance (Corbeanu et al., 2023). Burnout is also especially exacerbated by stress from interpersonal relationships (Kubo & Tao, 1992). In interpersonal helping professions, performance relates to education, assistance, or care quality. Examples include negative teacher–student relationships (Yoon, 2002), nurses’ infection control (Cimiotti et al., 2012), and physicians’ medical errors (Tawfik et al., 2018). Among ECEC teachers, burnout and mental health problems negatively impact the frequency of educational activities (Trauernicht et al., 2023), provision of emotional support and verbal interactions (Groeneveld et al., 2012), quality of teamwork (Nislin et al., 2015), and relationships with students (Whitaker et al., 2015).

Furthermore, burnout predicts turnover behaviour (Willard-Grace et al., 2019) and turnover intention (Rahim & Cosby, 2016). Burnout and mental health problems are also associated with increased turnover intention among ECEC teachers (Heilala et al., 2022), which may lead to actual turnover behaviour (Matsuda & Hamada, 2025). Therefore, considering the significant negative impact of teachers’ burnout on students’ development as well as the importance of maintaining continuity and quality in childcare and early childhood education, preventing and mitigating burnout among ECEC teachers is an urgent issue.

### Relationship between stress and burnout, and related factors

Although stress does not always result in burnout, many studies have assumed that burnout worsens because of occupational stress (McCormick & Barnett, 2011; Pines & Keinan, 2005). Some findings have indicated a cyclical process in which emotional exhaustion is exacerbated by and also causes stress, whereas depersonalisation and reduced personal accomplishment were not attributed to stress (McManus et al., 2002). Thus, while stress and burnout are strongly related, the relation varies among the subscales.

For occupational stress, this study conceptualises stress based on the job demands–control–support model (Karasek & Theorell, 1990; Karasek Jr., 1979). First, job demands and workload are positively associated with burnout (Greenglass et al., 2001; Xiaoming et al., 2014), with a stronger association reported with emotional exhaustion than depersonalisation and reduced personal accomplishment (Alarcon, 2011).

Second, studies have shown job control to be negatively related to burnout or to buffer the effects of job demands on burnout (Day et al., 2017; Portoghese et al., 2014). Previous meta-analyses have indicated that job control has a stronger negative association with depersonalisation and reduced personal accomplishment than emotional exhaustion (Park et al., 2014).

Third, social support has been emphasised in reducing stress and burnout or changing the relationship between them (Etzion, 1984; Guglielmi & Tatrow, 1998; Woodhead et al., 2016). For example, a systematic review of social support’s influence on nurse burnout showed that support from supervisors and colleagues is negatively associated with burnout (Velando‐Soriano et al., 2020). Among social support factors, supervisor and coworker support was considered most important in the workplace context (Charoensukmongkol et al., 2016; Velando‐Soriano et al., 2020). Supervisor support is also more strongly related to outcomes such as stress and performance than coworker support (Jolly et al., 2021). Moreover, Viswesvaran et al., (1999) found that social support alleviates stress responses, suppresses perceived stressors, and moderates the relationship between stressors and stress responses. However, they found no support for a mediating effect of stressors on stress reactions.

The role of workplace characteristics and climate in stress and burnout has also been examined. Psychological climate at work has been viewed as an antecedent to stressors and stress (Hemingway & Smith, 1999) or a moderating variable between stressors and burnout (Wen et al., 2020). Team climate affects burnout among nurses (Cheng et al., 2013), workplace climate influences organisational support and burnout among medical residents (Appelbaum et al., 2019), school climate impacts burnout among teachers (Grayson & Alvarez, 2008), and organisational climate affects burnout among mental health service workers (Aarons & Sawitzky, 2006). Thus, the impact of workplace climate on burnout was confirmed.

Therefore, how ECEC teachers perceive their work environment and its atmosphere may significantly influence the pathway from stress to burnout. Clarifying this relationship can provide insights for individual-, organisational-, and environmental-level interventions.

### Psychological safety

In this study, the workplace’s psychological climate represents psychological safety, defined as ‘a shared belief that the team is safe for interpersonal risk taking’ (Edmondson, 1999, 350) or ‘a climate in which individuals are comfortable in expressing and being themselves’ (Edmondson, 2018, xvi). Psychological safety is associated with antecedents such as personality; positive relationships with leaders; job design characteristics including autonomy, interdependence, and role clarity; and supportive work conditions. It is also linked to work engagement, task performance, information sharing, citizenship behaviour, creativity, learning behaviour, commitment, and job satisfaction (Frazier et al., 2017).

Recent studies increasingly addressed the relationship between psychological safety and burnout (Swendiman et al., 2019), revealing a negative association between the two (de Guillebon et al., 2025). Psychological safety mediates the negative relationship between leadership and burnout among employees, nurses, and athletes (Li & Peng, 2022; Ma et al., 2021; Yu et al., 2024). Further, psychological empowerment is negatively related to burnout among university students (Zhou & Chen, 2021). Studies suggested that psychological safety is influenced by antecedents such as cognitive stress (Hebles et al., 2021), work design characteristics including autonomy, and supportive work environments (Edmondson, 1999; Frazier et al., 2017), and it plays a suppressive role in burnout (Ma et al., 2021; Yu et al., 2024; Zhou & Chen, 2021). Based on Hobfoll (1989) conservation of resources theory which posits that individuals are motivated to maintain and further accumulate their resources (i.e. material, personal, and social resources), lack of job resources—such as support, autonomy, and employment stability—may lead to a decrease in psychological safety and, through the loss of resources, cause negative outcomes such as stress reactions. This process can be understood as a health impairment pathway (Newman et al., 2017). These findings suggest that psychological safety may serve as a mediating variable in the relationship between occupational stress and burnout, such that occupational stress negatively influences burnout indirectly through its effect on psychological safety. However, most research on psychological safety has been conducted in Western countries (Kim et al., 2020), and empirical evidence from Japan remains limited.

### Study purpose and hypotheses

To meet this gap, this study examines the mediating function of psychological safety in the relation between occupational stress and burnout using longitudinal data from two time points. A review of preventive interventions for burnout showed that organisationally led interventions, including task management and supervisory guidance, are fewer in number and examined in less detail than individually led interventions such as coping skills training and social support utilisation (Awa et al., 2010). However, combined interventions are effective, suggesting that investigating burnout prevention among ECEC teachers from organisational and cultural perspectives is meaningful.

**Figure 1.**
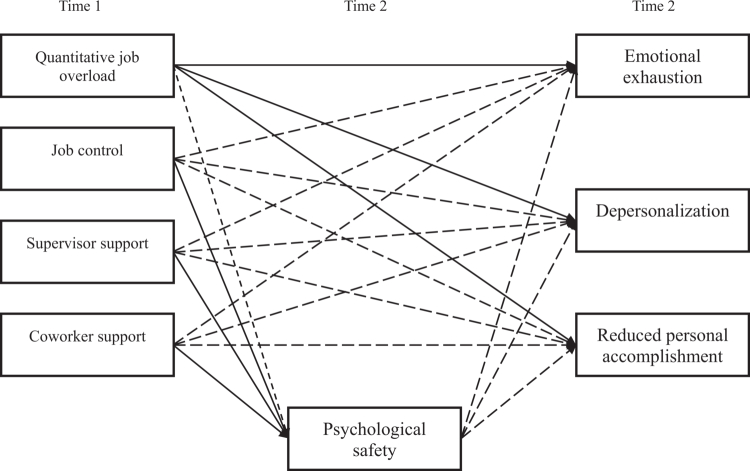
Hypothesised model in which psychological safety mediates the relationship between occupational stress and burnout. Note. Solid lines indicate positive effects; dashed lines indicate negative effects.

We hypothesise that ‘quantitative job overload’ measured at Time 1 is associated with increased burnout at Time 2, as reflected in its dimensions of ‘emotional exhaustion,’ ‘depersonalisation,’ and ‘reduced personal accomplishment.’ In contrast, we hypothesise ‘job control,’ ‘supervisor support,’ and ‘coworker support’ measured at Time 1 is associated with reduced burnout at Time 2. Additionally, we hypothesise that psychological safety measured at Time 2 mediates this relationship. Figure 1 presents the hypothesised model.

Based on the above, we formulate the following hypotheses:

Hypothesis 1: Higher levels of quantitative job overload is directly associated with higher levels of burnout, with the association with emotional exhaustion being stronger than that with the other two burnout dimensions. Specifically, the largest significant standardised regression coefficient is expected to be observed for emotional exhaustion.

Hypothesis 2: Higher levels of job control is associated with lower levels of burnout, with the association with emotional exhaustion being weaker compared to that of the other two dimensions. Specifically, the smallest or nonsignificant standardised regression coefficient is expected to be observed for emotional exhaustion.

Hypothesis 3: Higher levels of supervisor support and coworker support are directly associated with lower levels of burnout, with supervisor support exerting a stronger association than coworker support. Specifically, the standardised regression coefficient for supervisor support is expected to be larger than that for coworker support.

Hypothesis 4: Higher level of quantitative job overload is associated with higher level of burnout mediated by psychological safety.

Hypothesis 5: Higher level of job control is associated with lower levels of burnout mediated by psychological safety.

Hypothesis 6: Higher levels of supervisor and coworker support are associated with lower level of burnout mediated by psychological safety.

## Method

### Participants and procedure

An internet research company was used for data collection from registered monitors who completed a Japanese-language questionnaire. Participants were required to be currently working as ECEC teachers at a nursery centre, kindergarten, or certified child centre in Japan as an inclusion criterion. The Time-1 survey was conducted in early May 2023, yielding responses from 1,033 currently employed ECEC teachers. The Time-2 survey was conducted in late March 2024, resulting in 500 responses. However, 22 respondents were excluded because they had left their ECEC positions (19 individuals) or their responses were incomplete (three individuals). Consequently, data from 478 currently employed ECEC teachers were included in the final analysis (455 female individuals, 23 male individuals; mean age 37.14 ± 9.849 years; 310 nursery teachers, 37 kindergarten teachers, 117 certified childcare centre teachers, and 14 others). Among these 478 participants, none changed workplaces between Times 1 and 2. This study was not preregistered. Therefore, the analyses should be considered exploratory.

### Ethics statement

The first page of the online survey explained the purpose of the research, confidentiality, and the voluntary nature of participation. Only those who read and consented to cooperate could proceed to the survey. This study was approved by the Ethics Committee of the Faculty of Human Sciences, University of Tsukuba (approval number: Tsukuba 2023-222A; approval date: February 15, 2024).

### Measurements

#### Psychological safety at the workplace

The seven-item Japanese version of the Psychological Safety Scale developed by Edmondson (1999) was used, translated, and back-translated by Maruyama and Fuji (2022). An example item is, ‘It is safe to take a risk on this team.’ Higher scores indicate higher psychological safety. The Cronbach’s alpha in Maruyama and Fuji (2022) was .80. Participants responded on a seven-point Likert scale, indicating how they felt about their workplace and team.

#### Occupational stress

From the 23-item Brief Job Stress Questionnaire (Ministry of Health et al., 2015), factors that were considered causes of stress were measured: ‘quantitative job overload’ (three items; e.g. ‘I have a considerable amount of work to do’), ‘job control’ (three items; e.g. ‘I can work at my own pace’), and ‘supervisor support’ and ‘coworker support’ (three items each; e.g. ‘How freely can you talk with the following individuals?’). Items were rated on a four-point Likert scale. Higher scores for quantitative job overload indicate greater workload; higher scores for job control indicate greater discretion; higher scores for supervisor and coworker support indicate greater perceived support. This questionnaire is recommended for use in workplaces under the stress check guidelines of the Industrial Safety and Health Act (Ministry of Health et al., 2015).

#### Burnout

The Japanese version of Kubo and Tao (1994) burnout scale, comprising 17 items, was used. This scale was translated from the Maslach Burnout Inventory (Maslach & Jackson, 1981) and modified by deleting and adding items to suit interpersonal helping settings in Japan. It consists of three subscales rated on a five-point scale: ‘emotional exhaustion’ (five items; e.g. ‘Sometimes I want to quit this kind of work’), ‘depersonalisation’ (six items; e.g. ‘Sometimes I feel bothered by having to pay close attention to details’), and ‘personal accomplishment’ (six items; e.g. ‘I sometimes get so absorbed in my work that I lose track of time’). Initially developed for nurses, wording such as ‘colleagues and patients’ was replaced with ‘workplace stakeholders (colleagues, children, parents, etc.)’ in this study. All subscales were scored so that higher values indicate higher burnout.

### Data analysis

First, correlation coefficients were computed to clarify relations between scales. Next, covariance structure analysis was conducted based on the hypothesised model, specifying paths among variables, covariances among the four occupational stress variables, and covariances among the three burnout variables. Non-significant paths were removed to find the best-fitting model. Model fit criteria were goodness-of-fit index (GFI) ≥ .95, comparative fit index (CFI) ≥ .95, and root mean square error of approximation (RMSEA) ≤ .05.

To test the mediating effect of psychological safety, the bootstrap method (bias-corrected, 2,000 bootstrap samples, and 95% confidence interval [CI]) was used to examine the significance of indirect effects and calculate standardised indirect effects and bootstrap CIs. Total effects were also computed. To evaluate whether the sample size was sufficient to detect the hypothesised mediation effect, a Monte Carlo simulation was conducted using Mplus Version 8.11 (Muthén & Muthén, Los Angels, CA, USA). All other analyses were conducted using IBM SPSS Statistics 29.0 (IBM Corp., Armonk, NY, USA) and IBM SPSS Amos 28.0 (IBM Corp., Armonk, NY, USA). The significance level was set at 5%.

Following conventional notation in mediation analysis, the effect of occupational stress on psychological safety was denoted as path a, the effect of psychological safety on burnout was denoted as path b, and the direct effect of occupational stress on burnout was denoted as path c′.

In the population model, a small direct effect (c′ = .10) was specified alongside the indirect pathways. When small effect sizes (a = .10, b = .20) were assumed, the indirect effect was small (ab = .02), and the power to detect the indirect effect was approximately .55, indicating that the power may be limited.

In contrast, when moderate effect sizes (a = .20, b = .30) were assumed, the indirect effect increased (ab = .06), and statistical power exceeded .98.

These results suggest that the present sample size (*N* = 478) provides adequate statistical power to detect moderate mediation effects, even when controlling for the direct effect (c′). However, the power to detect small indirect effects may be limited and should be considered when interpreting non-significant findings.

## Results

### Correlation analysis between variables

Before testing the model, correlation coefficients among the variables were calculated (Table 1). All variables showed significant correlations except for quantitative job overload at Time 1. Quantitative job overload showed a weak negative correlation with job control (*r* = −.123, *p* < .01) and a moderate positive correlation with emotional exhaustion (*r* = .269, *p* < .001).

**Table 1.** Results of the correlation analysis among occupational stress at Time 1 and psychological safety and burnout at Time 2.

		Time 1								Time 2					
		Quantitative job overload		Job control		Supervisor support		Coworker support		Psychological safety		Emotional exhaustion		Depersonalisation	
Time 1	Job control	−.123**		–		–		–		–		–		–	
	Supervisor support	−.087		.403***		–		–		–		–		–	
	Coworker support	−.006		.302***		.585***		–		–		–		–	
Time 2	Psychological safety	.000		.332***		.490***		.415***		–		–		–	
	Emotional exhaustion	.269***		−.275***		−.260***		−.169***		−.404***		–		–	
	Depersonalisation	.062		−.275***		−.303***		−.298***		−.523***		.677***		–	
	Reduced personal accomplishment	−.034		−.296***		−.283***		−.233***		−.277***		.311***		.261***	

^**^ *p* < .01,

^***^ *p* < .001.

### Covariance structure analysis

**Figure 2.**
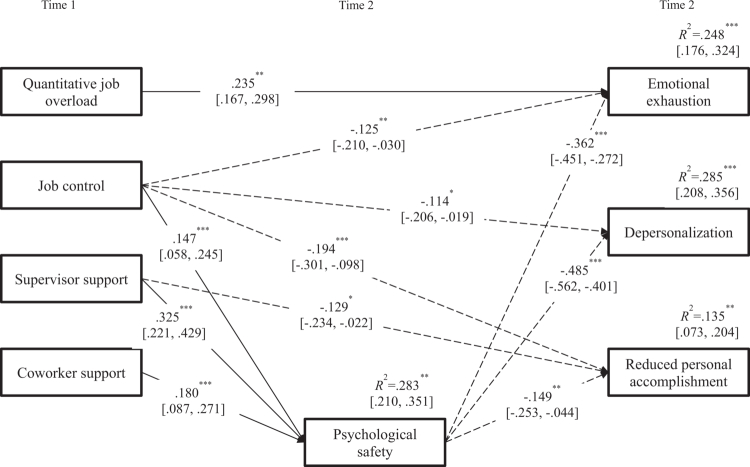
Results of the structural equation model showing the mediating effect of psychological safety on the relationship between occupational stress and burnout. Note. ^*^p < .05, ^**^p < .01, ^***^p < .001. Numbers represent standardised path coefficients; values in square brackets indicate 95% confidence intervals.

Following the hypothesised saturated model, covariance structure analysis was conducted with specified paths among variables. Non-significant paths were removed, and the final results are shown in Figure 2. The model demonstrated good fit: *χ*² = 16.909, *df* = 8, *p* = .031, GFI = .991, AGFI = .961, CFI = .991, and RMSEA = .048.

Quantitative job overload had a positive association only with emotional exhaustion (*β* = .235, *p* < .01, 95% CI [.167, .298]). Higher job control was associated with lower levels of all burnout subscales (emotional exhaustion: *β* = −.125, *p* < .01, 95% CI [−.210, −.030]; depersonalisation: *β* = −.114, *p* < .05, 95% CI [−.206, −.019]; reduced personal accomplishment: *β* = −.194, *p* < .01, 95% CI [−.301, −.098]) and as well as with higher levels of psychological safety (*β* = .147, *p* < .001, 95% CI [.058, .245]). Higher supervisor support was associated with a lower level of reduced personal accomplishment (*β* = −.129, *p* < .05, 95% CI [−.234, −.022]) and a higher level of psychological safety (*β* = .325, *p* < .001, 95% CI [.221, .429]). Higher coworker support was associated only with higher level of psychological safety (*β* = .180, *p* < .001, 95% CI [.087, .271]).

Higher psychological safety was associated with lower levels of all burnout subscales (emotional exhaustion: *β* = −.362, *p* < .01, 95% CI [−.451, −.272]; depersonalisation: *β* = −.485, *p* < .001, 95% CI [−.562, −.401]; reduced personal accomplishment: *β* = −.149, *p* < .01, 95% CI [−.253, −.044]).

### Mediation analysis

To confirm the mediating effect of psychological safety, the bootstrap method (bias-corrected, 2000 bootstrap samples, and 95% CI) was applied to test the significance of indirect effects and calculate standardised indirect effects and CIs (Table 2).

**Table 2.** Total and indirect effects of occupational stress on burnout mediated by psychological safety.

		Total effects						Indirect effects (mediated by psychological safety)					
						95%CI						95%CI	
Independent variables	Dependent variables	*B*	*SE*	*β*	*p*	LL	UL	*B*	*SE*	*β*	*p*	LL	UL
Quantitative job overload	Emotional exhaustion	.527	.074	.235	.002	.167	.298	―	―	―	―	―	―
Job control	Emotional exhaustion	−.421	.112	−.179	.002	−.264	−.079	−.125	.044	−.053	.001	−.095	−.022
	Depersonalisation	−.528	.150	−.185	.001	−.287	−.084	−.203	.070	−.071	.001	−.121	−.028
	Reduced personal accomplishment	−.528	.126	−.216	.001	−.321	−.116	−.054	.028	−.022	.004	−.053	−.006
Supervisor support	Emotional exhaustion	−.245	.054	−.118	.001	−.174	−.073	−.245	.054	−.118	.001	−.174	−.073
	Depersonalisation	−.397	.075	−.158	.001	−.225	−.106	−.397	.075	−.158	.001	−.225	−.106
	Reduced personal accomplishment	−.383	.106	−.178	.001	−.276	−.084	−.105	.042	−.049	.006	−.093	−.016
Coworker support	Emotional exhaustion	−.143	.041	−.065	.001	−.108	−.032	−.143	.041	−.065	.001	−.108	−.032
	Depersonalisation	−.232	.063	−.087	.001	−.139	−.043	−.232	.063	−.087	.001	−.139	−.043
	Reduced personal accomplishment	−.061	.028	−.027	.005	−.056	−.008	−.061	.028	−.027	.005	−.056	−.008
Psychological safety	Emotional exhaustion	−.245	.030	−.362	.001	−.451	−.272	―	―	―	―	―	―
	Depersonalisation	−.397	.034	−.485	.001	−.562	−.401	―	―	―	―	―	―
	Reduced personal accomplishment	−.105	.037	−.149	.009	−.253	−.044	―	―	―	―	―	―

Note: SE: standard error CI: confidence interval; LL: lower limit; UL: upper limit.

The results indicated significant indirect associations of job control (emotional exhaustion: *β* = −.053, *p* < .01, 95% CI [−.095, −.022]; depersonalisation: *β* = −0.071, *p* < .01, 95% CI [−.121, −.028]; reduced personal accomplishment: *β* = −.022, *p* < .01, 95% CI [−.053, −.006]), supervisor support (emotional exhaustion: *β* = −.118, *p* < .01, 95% CI [−.174, −.073]; depersonalisation: *β* = −.158, *p* < .01, 95% CI [−.225, −.106]; reduced personal accomplishment: *β* = −.049, *p* < .01, 95% CI [−.093, −.016]), and coworker support (emotional exhaustion: *β* = −0.065, *p* < .01, 95% CI [−.108, −.032]; depersonalisation: *β* = −.087, *p* < .01, 95% CI [−.139, −.043]; reduced personal accomplishment: *β* = −.027, *p* < .01, 95% CI [−.056, −.008]) with burnout through psychological safety. The total effects were also significant across all variables (|*β*| = .027 to .485).

## Discussion

This study aimed to examine the mediating role of psychological safety in the relation between occupational stress and burnout using longitudinal data collected at two time points. Hypotheses 1–3, concerning direct effects, and Hypotheses 4–6, concerning indirect effects, are discussed in sequence below.

### Hypotheses regarding the direct association of occupational stress with burnout

First, Hypothesis 1 was partially supported; quantitative job overload did not lead to increased depersonalisation or reduced personal accomplishment. However, it was positively and significantly associated with emotional exhaustion, indicating higher levels of emotional exhaustion, which is consistent with expectations and prior research (Greenglass et al., 2001; Janssen et al., 1999).

Hypothesis 2 was partially supported; higher job control was associated with lower level of all burnout dimensions both directly and overall, but the association with emotional exhaustion was not notably smaller than the other dimensions. This is in contrast with other previous findings: for early childhood teachers in the United States, job control was related to all three burnout dimensions but had the most potent association with emotional exhaustion (Schaack et al., 2020), suggesting possible differences by profession or country.

In this study, the total effect of job control was relatively larger for reduced personal accomplishment (*β* = −.216), with a direct considerable impact (*β* = −.199), suggesting that enhancing job control may be especially effective for improving personal accomplishment. Meta-analyses on job control and burnout indicate stronger correlations with depersonalisation and personal accomplishment than with emotional exhaustion, with personal accomplishment (*ρ*^ = .36) having a stronger association than depersonalisation (*ρ*^ = −.29; Park et al., 2014). Individual accomplishment is closely linked to self-efficacy (Aloe et al., 2014; Shoji et al., 2016). Thus, being entrusted with discretion and making independent decisions may enhance professional confidence.

The proportion of workers with low job control was 5.5% for women and 5.4% for men in the general workforce (Ministry of Health et al., 2015), compared to 3.4% for women and 10.2% for men among ECEC teachers (Matsuda & Hamada, 2024). As female ECEC teachers appear less exposed to low job control, they may be less likely to experience its negative impacts; accordingly, job control may become an important protective factor for this group.

Hypothesis 3 was partially supported; only supervisor support demonstrated a significant direct negative association with burnout, whereas coworker support was not significantly associated with burnout. Thus, as expected, the direct association between supervisor support and burnout was stronger than that between coworker support and burnout.

### Hypotheses regarding the indirect association of psychological safety with burnout

Hypothesis 4 was not supported because the association between quantitative job overload and burnout was not mediated by psychological safety. According to the Ministry of Health et al. (2015), 5.8% and 10.4% of women and men, respectively, scored high on quantitative job overload. In contrast, for ECEC teachers, the percentages were 19.5% and 16.1% for women and men, respectively (Matsuda & Hamada, 2024). These findings indicate that ECEC teachers widely experience high job demands and strong emotional exhaustion. Therefore, a high quantitative workload may be a common experience across workplaces and may not be directly related to the sense of psychological safety within the team or workplace relations.

Furthermore, Hypothesis 5 was supported; job control partially mediated the association with emotional exhaustion, depersonalisation, and reduced personal accomplishment via psychological safety. This finding suggests that increasing discretion at work benefits individual mental health and the perception of interpersonal safety within the workplace and team.

Finally, Hypothesis 6 was supported; higher levels of supervisor and coworker support were associated with lower level of burnout and mediated by psychological safety. The total effects indicated that supervisor support was more effective than coworker support in reducing burnout. Supervisor support also directly diminished the sense of reduced personal accomplishment, implying that such support efficiently enhances competence and feeling of achievement among ECEC teachers. For example, transformational leadership has been shown to reduce burnout partially through self-efficacy (Tian & Guo, 2024), suggesting that supervisors’ support positively influences supervisees’ motivation, attitudes, and emotions, thereby suppressing burnout.

Except for the association of supervisor support with reduced personal accomplishment, all relationships between support and burnout were fully mediated by psychological safety. This result indicates that social support does not directly suppress burnout but significantly enhances psychological safety.

However, Schaack et al. (2020) found that leadership did not affect burnout, while collegiality negatively influenced emotional exhaustion and depersonalisation, and indirectly negatively affected turnover intention via emotional exhaustion. Based on this finding, coworker relations might be especially critical for turnover.

### Implications for practice and significance of this study

Psychological safety has been widely studied as a factor enhancing workplace performance, such as creativity (Maruyama & Fuji, 2022; Yang et al., 2021), voice behaviour (O’Donovan & McAuliffe, 2020), and learning behaviour (Kim et al., 2020). Our study supports its relevance to individual mental health, particularly indicating a potent suppressive effect on depersonalisation. These results suggest that psychological safety is essential for mental health care for ECEC teachers. The importance of supervisors’ leadership in enhancing psychological safety has also been noted (Nembhard & Edmondson, 2006), consistent with the present findings. Given that supervisors can also manage quantitative job overload and job control, systematic interventions by administrators are needed to prevent burnout in ECEC teachers.

Additionally, burnout and stress factors mutually influence each other; however, burnout exacerbates stress factors more strongly than stress factors exacerbate burnout (Guthier et al., 2020). Building psychological safety could help break this vicious cycle. Research on psychological safety in Japan is still developing, and this study expands knowledge in the mental health domain, representing a significant contribution.

Conversely, emotional exhaustion was directly associated with quantitative job overload. Emotional exhaustion is a core symptom of burnout (Seidler et al., 2014). It is reported to precede depersonalisation and reduce personal accomplishment (Shih et al., 2013), underscoring the importance of interventions targeting quantitative job overload of ECEC teachers.

### Limitations

This study has three main limitations. First, the participants were monitors registered with an internet survey company, so caution must be applied in generalising the findings to the broader population of ECEC teachers. Among those who responded at Time 2, 19 had already left the ECEC field. Therefore, future studies should aim to collaborate directly with ECEC facilities and adopt research designs that allow for follow-up with individuals who have left the field.

Second, cultural influences should be considered when interpreting the findings of this study. Although research on psychological safety has primarily been conducted in Western cultural contexts, Newman et al. (2017) argue that differences in its function across cultures should also be taken into account. In Eastern cultural contexts, including Japan, where harmony is highly valued, individuals tend to experience greater hesitation in expressing their thoughts and emotions. Therefore, the effects of perceived psychological safety may differ from those observed in Western cultures (Ochiai & Otsuka, 2022). Notably, because this study focused on Japanese ECEC teachers, culture-specific factors in Japan may have influenced the results.

Third, psychological safety and burnout were measured simultaneously, making causal inference between them insufficient. Higher burnout may impair perception of psychological safety, and further longitudinal studies are needed to clarify this. Specifically, panel surveys on psychological safety and burnout must be conducted to better clarify their reciprocal relation.

## Conclusion

This study aimed to examine the mediating role of psychological safety in the relation between occupational stress and burnout among ECEC teachers using longitudinal data from two time points. Covariance structure analysis showed that quantitative job overload exacerbated emotional exhaustion, job control suppressed all burnout dimensions, and supervisor support reduced only personal accomplishment. Mediation analysis revealed that psychological safety partially mediated the association of job control with burnout and of supervisor support with reduced personal accomplishment, and fully mediated the association of supervisor support with emotional exhaustion and depersonalisation, including coworker support in all burnout dimensions. These findings confirm the necessity of considering psychological safety in the relation between occupational stress and burnout, providing a novel perspective for constructing mental health measures for ECEC teachers.

## Data Availability

The data supporting this study’s findings are available from the corresponding author, Yuko Matsuda, upon reasonable request. This study is a reanalysis of Matsuda and Hamada (2025) study, which was presented at the 89th Annual Convention of the Japanese Psychological Association. The Time 1 data used in the present study were also used in Matsuda and Hamada’s work, which aimed to identify predictors of turnover among ECEC teachers. Despite using the same dataset, the previous and present study are entirely independent regarding research objectives and analytical focus. The corresponding author takes responsibility for the integrity of the data and the accuracy of the data analysis. Informed consent was obtained from all participants involved in the study. The study was conducted in accordance with the Declaration of Helsinki and was approved by the Ethics Committee of the Faculty of Human Sciences, University of Tsukuba (approval number: Tsukuba 2023-222A; approval date: February 15, 2024). See details under Methods.
